# Evidence for Arrhythmogenic Effects of A_2A_-Adenosine Receptors

**DOI:** 10.3389/fphar.2019.01051

**Published:** 2019-09-18

**Authors:** Peter Boknik, Katharina Drzewiecki, John Eskandar, Ulrich Gergs, Britt Hofmann, Hendrik Treede, Stephanie Grote-Wessels, Larissa Fabritz, Paulus Kirchhof, Lisa Fortmüller, Frank Ulrich Müller, Wilhelm Schmitz, Norbert Zimmermann, Uwe Kirchhefer, Joachim Neumann

**Affiliations:** ^1^Institut für Pharmakologie und Toxikologie, Universitätsklinikum Münster, Westfälische Wilhelms-Universität, Münster, Germany; ^2^Institut für Pharmakologie und Toxikologie, Medizinische Fakultät, Martin-Luther-Universität Halle-Wittenberg, Halle, Germany; ^3^Klinik für Herzchirurgie, Medizinische Fakultät, Martin-Luther-Universität Halle-Wittenberg, Halle, Germany; ^4^Institute of Cardiovascular Sciences, University of Birmingham, Birmingham, United Kingdom; ^5^University Hospitals Birmingham NHS Foundation Trust, Birmingham, United Kingdom; ^6^Sandwell and West Birmingham Hospital NHS Trust, Birmingham, United Kingdom; ^7^Institute for Human Genetics, Genetic epidemiology, Universitätsklinikum Münster, Westfälische Wilhelms-Universität, Münster, Germany; ^8^Bundesinstitut für Arzneimittel und Medizinprodukte, Bonn, Germany

**Keywords:** A_2A_-adenosine receptor, contractility, ischemia, reperfusion, arrhythmias, human heart

## Abstract

Adenosine can be released from the heart and may stimulate four different cardiac adenosine receptors. A receptor subtype that couples to the generation of cyclic adenosine monophosphate (cAMP) is the A_2A_-adenosine receptor (A_2A_-AR). To better understand its role in cardiac function, we studied mechanical and electrophysiological effects in transgenic mice that overexpress the human A_2A_-AR in cardiomyocytes (A_2A_-TG). We used isolated preparations from the left atrium, the right atrium, isolated perfused hearts with surface electrocardiogram (ECG) recording, and surface body ECG recordings of living mice. The hypothesized arrhythmogenic effects of transgenicity per se and A_2A_-AR stimulation were studied. We noted an increase in the incidence of supraventricular and ventricular arrhythmias under these conditions in A_2A_-TG. Moreover, we noted that the A_2A_-AR agonist CGS 21680 exerted positive inotropic effect in isolated human electrically driven (1 Hz) right atrial trabeculae carneae. We conclude that A_2A_-ARs are functional not only in A_2A_-TG but also in isolated human atrial preparations. A_2A_-ARs in A_2A_-TG per se and their stimulation can lead to cardiac arrhythmias not only in isolated cardiac preparations from A_2A_-TG but also in living A_2A_-TG.

## Introduction

Adenosine is well known to elicit cardiac arrhythmias. In their famous paper that founded the field of adenosine pharmacology, Drury and Szent-Györgyi (1929) showed that adenosine can induce arrhythmias, namely, bradycardia. For instance, adenosine has a negative chronotropic effect on the sinus node and a negative dzromotropic effect on the AV-node (Shryock and Belardinelli, 1997). Adenosine can interact with A_1_-, A_2A_-, A_2B_-, and A_3_-adenosine receptors (AR). Typically, A_1_- and A_3_-AR inhibit and A_2A_- and A_2B_-AR stimulate adenylyl cyclase activities (for review, see Olsson and Pearson, 1990). From all P_1_-purinoceptors, the A_2A_-AR was cloned first (Libert et al., 1989).

Cardiomyocytes are able to synthesize and release adenosine. Moreover, cardiac adenosine release is markedly stimulated by β-adrenoceptor activation, ischemia, or necrosis of the heart. Clinically, adenosine and adenosine triphosphate (ATP), the precursor of adenosine, are useful to treat supraventricular tachyarrhythmias. Hence, in the human heart, the actions of adenosine are of clinical relevance and merit further investigation. If A_2A_-ARs are in general functionally (increase in cAMP, contractility, and heart rate) expressed in the mammalian heart is controversially discussed. Species- or method-dependent differences may exist: in some publications, no cardiac effects of adenosine were reported (rat: Shryock et al., 1993, guinea-pig: Boknik et al., 1997, rabbit: Kilpatrick et al., 2002), whereas in others, a functional cardiac response to adenosine was noted (mouse: Morrison et al., 2006, rat: Monahan et al., 2000).

In human hearts, A_2A_-ARs have been detected on the protein level (Marala and Mustafa, 1998, Hove-Madsen et al., 2006). Moreover, by use of mice with constitutive deletion of A_2A_-ARs (A_2A_-AR-KO mouse), the functional expression of A_2A_-ARs in the wild-type (WT) mouse heart could be demonstrated (Ledent et al., 1997). Ledent et al. compared isolated perfused hearts from WT and A_2A_-AR-KO mice and clearly established that CGS 21680, a selective A_2A_-AR agonist, increased contractility only in WT hearts but not in A_2A_-AR-KO hearts (Ledent et al., 1997). Furthermore, it has been shown that stimulation of A_2A_-AR can protect the heart from reperfusion damage (e.g. rabbit: Cargnoni et al., 1999).

More recently, A_2A_-ARs were shown to be expressed in human atrial preparations and this may lead to alterations in the frequency of spontaneous Ca^2+^ release (Hove-Madsen et al., 2006). Alternatively, the action of adenosine to induce bradycardia may subsequently lead to atrial fibrillation (Isa-Param et al., 2006). Ischemia induced increases in adenosine might trigger arrhythmias (Bertolet et al., 1997).

Interestingly, atrial fibrillation in patients was accompanied by an increase of A_2A_-ARs on mRNA and protein level in atrial tissue and an increase in cardiac ryanodine receptor (RYR2) phosphorylation, and this was suggested to lead to an altered flow of Ca^2+^ through the sarcolemmal sodium calcium exchanger (NCX) and thus arrhythmias (Llach et al., 2011a). The A_2A_-AR agonist CGS 21680 enhanced currents through NCX in isolated atrial cardiomyocytes from patients with atrial fibrillation but not in samples from patients in sinus rhythm (Llach et al., 2011a). Also, endogenous adenosine stimulated currents through NCX in isolated atrial cardiomyocytes from patients with atrial fibrillation (Llach et al., 2011a). It has been suggested that A_2A_-AR stimulation may increase the Ca^2+^ content of the sarcoplasmic reticulum (SR) and NCX stimulation might increase Ca^2+^ inflow into the cell. This increase of Ca^2+^ content in the SR may lead to increased release of Ca^2+^ from the SR, leading to delayed afterdepolarization and thus to atrial arrhythmias like atrial fibrillation (Llach et al., 2011a).

In the present study, we tested the hypothesis that overexpression of A_2A_-ARs increases the susceptibility to arrhythmias in our recently described model of human A_2A_-AR overexpressing mice (Boknik et al., 2018) under basal or stimulated conditions. Furthermore, we studied the functional role of A_2A_-AR stimulation in isolated paced right atrial preparations from diseased human hearts in order to ascertain that A_2A_-ARs are functional in the human heart.

Our results provide evidence for a contractile effect of the stimulation of A_2A_-AR in human hearts and a proarrhythmic effect of A_2A_-ARs in normoxia (under basal and drug-stimulated conditions) and hypoxia in mammalian cardiac preparations. Progress reports of this work have appeared in abstract form (Neumann et al., 2017; Neumann et al., 2018).

## Materials and Methods

### Isolation of A_2A_-AR CDNA and Generation of Transgenic Mice

The generation of transgenic mice has been recently described (Boknik et al., 2018). The investigation conforms to the Guide for the Care and Use of Laboratory Animals published by the National Research Council (2011). Animals were handled and maintained according to approved protocols of the animal welfare committee of the University of Münster, Germany.

The polymerase chain reaction (PCR)-generated human A_2A_-A cDNA fragment containing a 3´ and 5´engineered NotI digestion site was inserted into a mouse cardiac α-myosin heavy chain promoter expression cassette. Genotypes were identified by PCR analyses of tail-tip DNA using the following primers: 5´-acaaagcaggcgatgaag-3´ and 5’-acccttaccccacatagacc-3´. Reverse transcription was performed with 4 µg RNA using random primers (Transcriptor High Fidelity cDNA Synthesis Kit, Roche Applied Science, Mannheim, Germany), and PCR amplification was performed using the Ampliqon Taq DNA polymerase (Biomol, Hamburg, Germany) according to the manufacturer’s instructions. All experiments presented here were performed on 12- to 30-week-old A_2A_-TG mice and WT littermates of both sexes.

### Western Blot Analysis

Cardiac homogenates were prepared in 300 µl of 10 mM NaHCO_3_ and 100 µl 20% SDS (sodium dodecyl sulfate). Mixtures were kept at 25°C for 30 min before centrifugation to remove debris. Thereafter, supernatants (called homogenates) were kept at −20°C until further analysis. Western blot analysis was performed as reported (Gergs et al., 2004). Aliquots of 100 µg of protein were loaded per lane. The antibodies against ERK (extracellular regulated kinase), AKT (protein kinase B), phospho-ERK, phospho-AKT, and A_2A_-ARs were obtained from Merck (Darmstadt, Germany). All secondary antibodies were conjugated with an alkaline phosphatase (Sigma-Aldrich, Taufkirchen, Germany). Bands were detected using enhanced chemifluorescence (GE Healthcare, Freiburg, Germany), and fluorescent bands were visualized in a Typhoon 9410 PhosphorImager and quantified using the ImageQuaNT software (GE Healthcare, Freiburg, Germany). Enhanced chemifluorescence detection was carried out according to the manufacturer’s instructions (GE Healthcare, Freiburg, Germany).

### Cardiomyocyte Isolation

Isolation of ventricular cardiomyocytes from A_2A_-TG and WT mice, measurement of Ca^2+^ transients, and measurement of cell shortening were performed as described previously (Kirchhefer et al., 2001). To avoid the interference from endogenous adenosine, adenosine deaminase (ADA) (5 units ml^−1^) was present under all experimental conditions. In order to get an insight into the signaling *via* A_2A_-AR, we used the following highly selective antagonists for pre-incubation: DPCPX (A_1_-AR antagonist) or ZM 241385 (A_2A_-AR antagonist). Isolated cardiomyocytes were stimulated either by 1 µM CGS 21680 (A_2A_-AR agonist) or by 1 µM isoproterenol (β-adrenergic stimulation as positive control) each for 10 min at 37°C.

### ECG Recordings in Awake A_2A_-TG Mice and WT Littermates

Adult (9 months old) mice were instrumented with a telemetric ECG transmitter (Data Science, Minneapolis, USA) as described previously (Kirchhof et al., 2003). After a postoperative recovery period, telemetric ECG recordings were obtained in freely moving mice continuously for 24 h. Besides the 24 h protocol, ECGs were recorded after i.p. injection of the A_2A_-AR agonist CGS 21680 (40 µg kg^−1^). Before injection, ECGs were recorded for 1 h to preserve basal conditions. ECGs were analyzed for heart rate and arrhythmias.

### Echocardiography

Echocardiography in spontaneously breathing mice was performed under anesthesia with 1.5% isofluorane as described previously (Fabritz et al., 2004; Gergs et al., 2010). We assessed the cardiac function and diameters under baseline and after i.p. injection of the beta-adrenoceptor agonist isoproterenol (2 mg kg^−1^).

### Contractile Function

Contractile function was studied as reported (Boknik et al., 2018; Gergs et al., 2018). In brief, mice were anesthetized by i.p. injection of pentobarbital sodium (50 mg kg^−1^) and hearts were excised. Pentobarbital was used here in order to provide direct comparability with our previous work (e.g. Boknik et al., 2018) and followed the suggestions of our animal protection committee. Right and left atria (about 3 mm of length) were dissected from isolated hearts and mounted in an organ bath. Left atrial preparations were continuously electrically stimulated (field stimulation was used for comparability with all our previous work: e.g. Kirchhefer et al., 2001; Gergs et al., 2013; Boknik et al., 2018) with each impulse consisting of 1 Hz, with a voltage of 10–15% above threshold and 5 ms duration. Right atrial preparations were allowed to contract spontaneously. The bathing solution contained (in mM) NaCI 119.8, KCI 5.4, CaCl_2_ 1.8, MgCl_2_ 1.05, NaH_2_PO_4_ 0.42, NaHCO_3_ 22.6, Na_2_EDTA 0.05, ascorbic acid 0.28, and glucose 5.0, continuously gassed with 95% O_2_ and 5% CO_2_, and maintained at 35°C resulting in a pH of 7.4. Signals detected *via* an isometric force transducer were amplified and continuously recorded. CGS 21680, ZM 241385, or isoproterenol (1 µM each) was cumulatively applied for 20 min for each compound. Contraction experiments were performed after addition of ADA (1 µg ml^−1^) and DPCPX (1 µM) to avoid interference from endogenous adenosine or A_1_-AR activation. For comparison, right atrial preparations were obtained from patients undergoing bypass surgery due to coronary heart disease and handled in the same way as left atrial preparations from mice (see Human Atrial Preparations).

### Langendorff-Perfused Hearts

Heart preparations were utilized as described previously (Boknik et al., 2018). Mice were anesthetized intraperitoneally with 2.0 g kg^−1^ body weight urethane and treated with 1.5 units of heparin. This was done to provide direct comparability with our previous work (e.g. Boknik et al., 2018) and followed the suggestions of our animal protection committee. The hearts were removed from the opened chest, immediately attached by the aorta to a 20-gauge cannula, and perfused retrogradely with oxygenized Krebs-Henseleit buffer (37.4 C) containing 118 mM NaCl, 25 mM NaHCO_3_, 0.5 mM Na-EDTA, 4.7 mM KCl, 1.2 mM KH_2_PO_4_, 1.2 mM MgSO_4_, 2.5 mM CaCl_2_, and 11 mM glucose in an isolated heart system (Hugo Sachs Elektronik, Freiburg, Germany). Hearts were stimulated at 8 Hz. Heart rate, aortic pressure, and LV pressure were measured and monitored continuously. The first derivative of LV pressure (+dP/dt and −dP/dt) was calculated (ADInstruments, Oxford, UK). In order to achieve global ischemia, the perfusion was stopped for 20 min and thereafter the hearts were perfused for 60 min. In an additional set of experiments, electrophysiological studies (recording of monophasic action potentials) were performed in isolated, Langendorff-perfused hearts as described previously (Kirchhof et al., 2007).

### Human Atrial Preparations

Right atrium (RA) samples were obtained from patients undergoing open-heart surgery with coronary artery bypass grafts and electrically stimulated in organ baths as described previously (Gergs et al., 2008; Gergs et al., 2013; Gergs et al., 2018). Patients were treated with the following drugs: acetyl salicylic acid (ASS), clopidogrel, bisoprolol, thyroxine, atorvastatin, pantoprazole, olmersartan, amlodipine, frusemide, metformin, rivaroxaban, ipratropiumbromide, fenoterol, simvastatin, torasemide, esomeprazole, flucatison, salmeterol, ramipril, and hydrochlorothiazide. Patients were in CCS (Canadian Cardiovascular Society, angina classification) scale from III to IV and NYHA (New York Heart Association, heart failure classification) class II–III. Left ventricular ejection fraction ranged from 40 to 55%. This study complied with the Declaration of Helsinki and was approved by the local ethics committee (hm-bü 04.08.2005). All patients gave informed consent.

### Data Analysis

Data shown are means ± SEM. Statistical significance was estimated by analysis of variance followed by Bonferroni´s t-test or using the χ^2^-test as appropriate. A p-value < 0.05 was considered as significant.

### Drugs and Materials

ADA (produced by Roche) was purchased from Sigma-Aldrich (#10102121001), CGS 21680 (2-p-(2-carboxyethyl)phenethylamino-5′-N-ethylcarboxamidoadenosine hydrochloride hydrate) was purchased from Sigma-Aldrich (#C141), DPCPX (8-cyclopentyl-1,3-dipropylxanthine) was purchased from TOCRIS (#0439), and ZM 241385 (4-(2-[7-amino-2-(2-furyl)[1,2,4]triazolo[2,3-a][1,3,5]triazin-5-ylamino]ethyl)phenol) was purchased from TOCRIS (#1036). All other chemicals were of analytical grade. Deionized water was used throughout the experiments. Stock solutions were freshly prepared daily.

## Results

**Figure 1:**
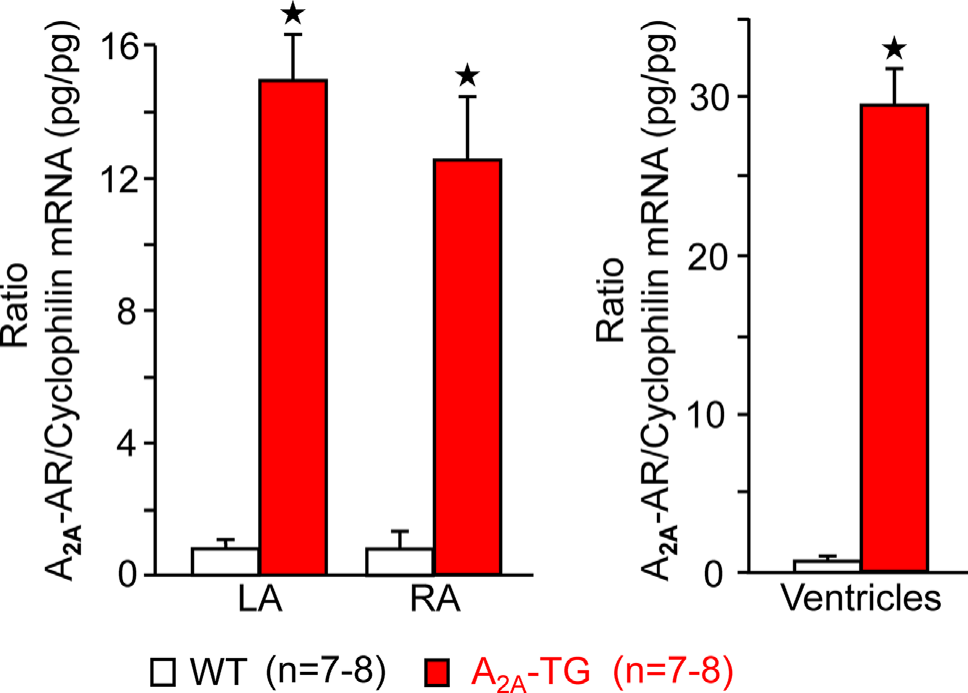
A_2A_-AR-mRNA is greatly enhanced in cardiac preparations from A_2A_-TG compared to WT. Assessment by means of quantitative RT-PCR of human A_2A_-ARs in left (LA) and right atria (RA) and ventricles from WT and A_2A_-TG. As internal control, the level of cyclophilin was also determined and the ratio is plotted here. ★*p* < 0.05 versus WT. Numbers in brackets indicate number of samples studied.

**Figure 2:**
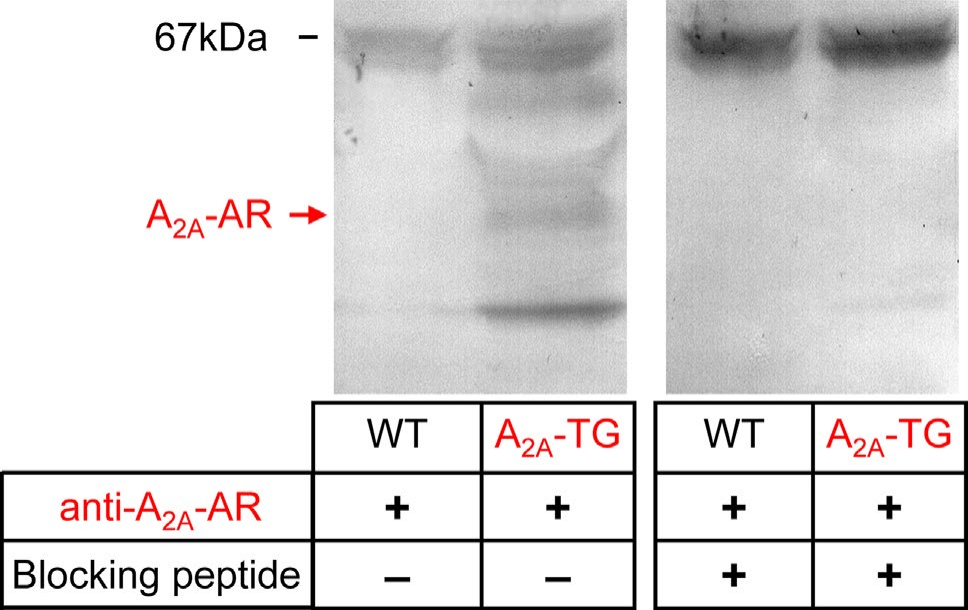
A_2A_-AR is increased at protein level in the heart of A_2A_-TG. Whole hearts from WT and A_2A_-TG were homogenized and subjected to electrophoresis, transferred to nitrocellulose membranes, and incubated with an antibody against the A_2A_-AR. The putative specific signals in A_2A_-TG lanes were blocked when the antibodies were pre-incubated with a blocking peptide. At 67 kDa, unspecific bands were located, while the specific band for monomeric A_2A_-AR is indicated by an arrow.

**Figure 3:**
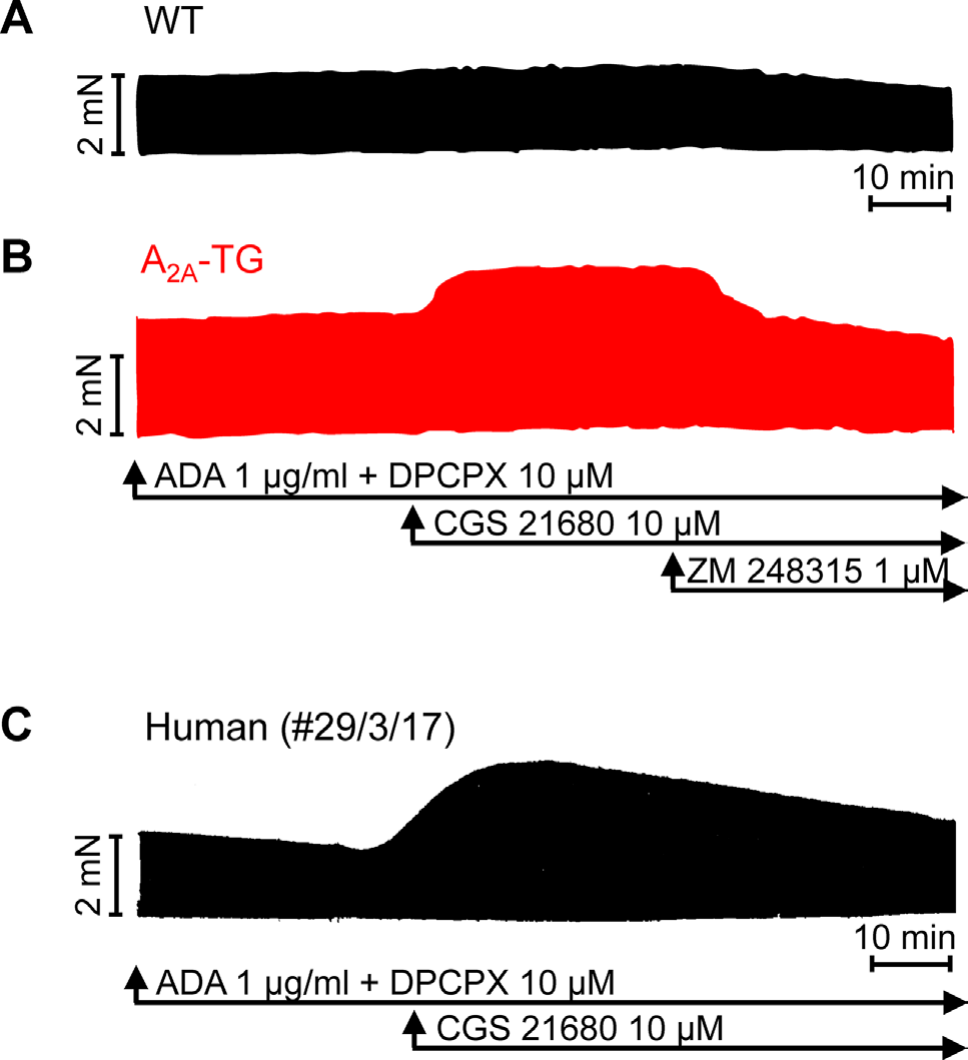
Stimulation of A_2A_-AR by CGS 21680 increased force contraction in the presence of adenosine deaminase (ADA) and an A_1_-AR antagonist (DPCPX) in atrial preparations from A_2A_-TG **(B)** and man **(C)** but not in WT **(A)**. The effects of CGS 21680 in A_2A_-TG **(B)** were blocked by the A_2A_-AR antagonist ZM 241385. Ordinates indicate force of contraction in mN (milli-Newton). Lengths of the horizontal bars indicate the time scale of the experiments.

For the interpretation of data on atrial samples, it was deemed important to show that overexpression in the atria occurs. To this end, we performed RT-PCR experiments. The expression of A_2A_-ARs was similar in RA as in left atrium (LA) of A_2A_-TG. However, expression in the ventricle of A_2A_-TG was about double to that of transgenic atria (**Figure 1**). There is evidence in the literature that mRNA is not always translated to protein; hence, it was of interest to study whether increase in mRNA for the A_2A_-AR is also reflected by increased levels of A_2A_-AR protein. Therefore, we next tried to detect the A_2A_-AR in ventricular samples on protein level using an antibody. We noted several bands in extracts from A_2A_-TG hearts (**Figure 2**, second lane from the left) that were absent in samples from WT but were blocked by a peptide (to which the antibody had been generated). We are not sure how to interpret the several bands in A_2A_-TG samples (see Discussion). For lack of sufficient amount of tissue, we could not perform Western blots in atrial preparations; however, we could present functional data on the presence of A_2A_-AR in atrial samples (**Figure 3**). As seen in the middle lane (of Fig: 3) CGS 21680, an agonist at A_2A_-ARs increased force of contraction in isolated left atrial preparations from A_2A_-TG, but this positive inotropic effect (PIE) was lacking in left atrial preparations from WT (in agreement with our previous work: Boknik et al., 2018). This PIE was blocked by addition of the A_2A_-AR ZM 241385. A novel finding is, however, that using the same experimental conditions, we detected a PIE to CGS 21680 in electrically driven preparations from human RA (bottom lane in **Figure 3**) and this amounted to 119 ± 12% (p < 0.05, nine trabeculae carneae from five patients undergoing bypass surgery due to coronary heart disease).

**Figure 4:**
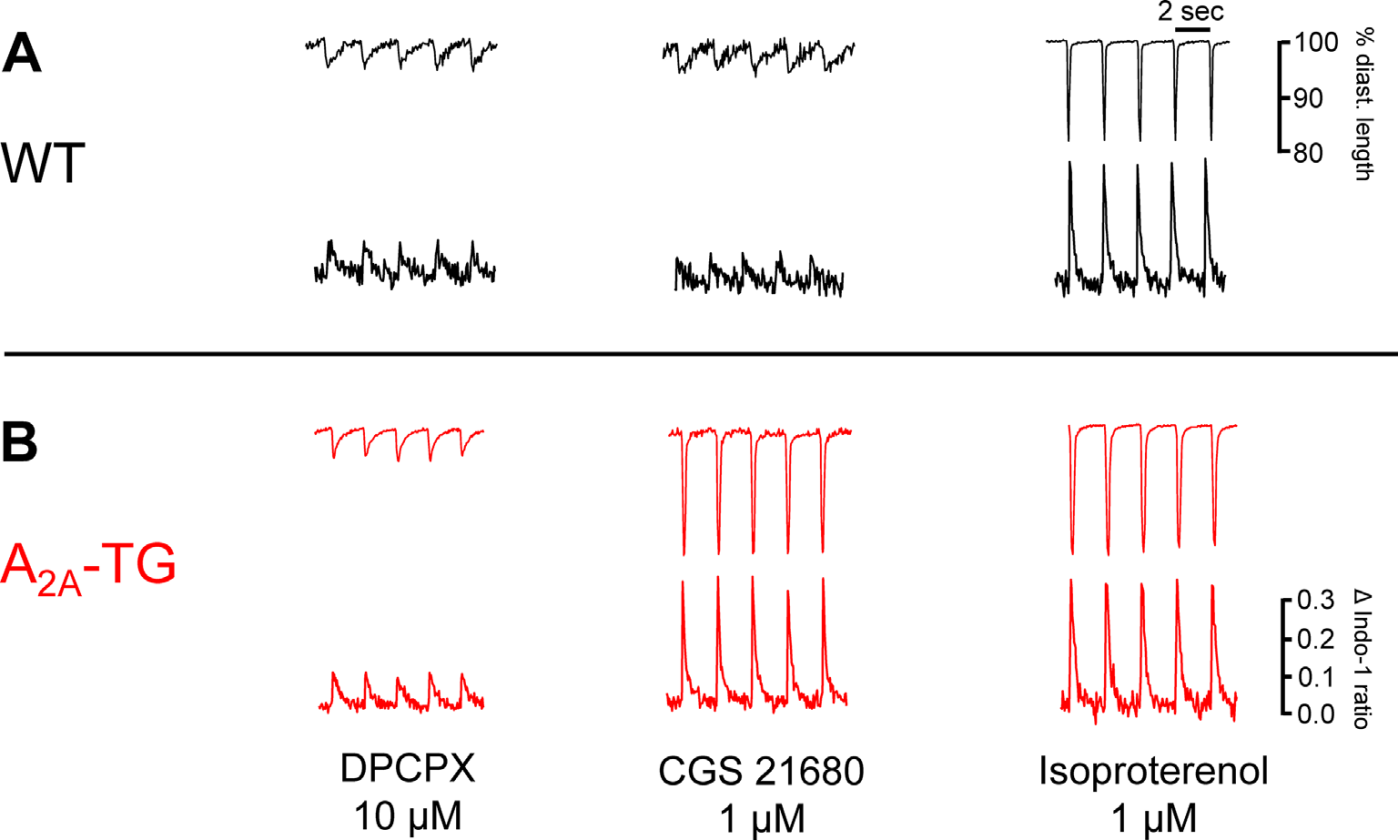
The A_2A_-AR agonist CGS 21680 increased cytosolic Ca^2+^ and cell shortening only in A_2A_-TG cardiomyocytes **(B)** but not in WT cardiomyocytes **(A)**. Isolated ventricular cardiomyocytes were stained with a Ca^2+^ sensitive dye (Indo-1) and electrically paced (1 Hz). The length of longitudinal cell motion (**A** with scale bars) and Ca^2+^ sensitive fluorescence are presented (**B** with scale bars). On the left hand side, cardiomyocytes were incubated with 10 µM DPCPX and 1 µg ml^−1^ ADA; in the middle, the effects of additionally applied CGS 21680 (1 µM) are shown. The effects of additionally applied 1 µM isoproterenol were comparable in WT cardiomyocytes and A_2A_-TG cardiomyocytes (right hand side **A** versus **B**).

Another new mechanistic finding is that the PIE of CGS 21680 in A_2A_-TG was accompanied by an increase in intracellular Ca^2+^ transients and increased cell shortening (i.e. contractility) in ventricular cardiomyocytes from A_2A_-TG but not WT (**Figure 4**). As a positive control, we noted that the β-adrenoceptor agonist isoproterenol increased both intracellular cytosolic Ca^2+^ content and cell shortening in isolated ventricular cardiomyocytes from both WT and A_2A_-TG (**Figure 4**, right hand side). Echocardiographic measurements showed increased diastolic diameter of left atria from A_2A_-TG (**Table 1**). Doppler measurements of blood flow through the mitral valve (MV) showed higher pressure and velocities in hearts from A_2A_-TG compared to hearts from WT (**Table 1**). After injection of isoproterenol, both genotypes developed similar values (**Table 1**). Left ventricular ejection fraction (a parameter of cardiac contractility) and left ventricle diameters were not altered under basal conditions, but after injection of isoproterenol, contractility in hearts from A_2A_-TG was lower as compared to WT (**Table 1**). In 4 of 15 A_2A_-TG mice, spontaneous ventricular extrasystoles appeared, none in WT mice during echocardiography.

**Table 1:** Echocardiographic measurements under basal conditions (BASE) and after application of isoproterenol (ISO).

Parameters	BASE		ISO	
	A_2A_-TG	WT	A_2A_-TG	WT
Age (weeks)	15 ± 0.66	14 ± 0.74	15 ± 0.66	14 ± 0.74
N	15	15	15	15
HR (bpm)	467 ± 8	453 ± 9	635 ± 8	635 ± 8
LA (mm)	1.23 ± 0.03*	1.08 ± 0.01	1.21 ± 0.03*	1.07 ± 0.01
LVEDd (mm)	3.78 ± 0.08	3.58 ± 0.07	3.18 ± 0.12*	2.87 ± 0.06
EDV (µl)	61.79 ± 2.95	54.15 ± 2.50	41.50 ± 3.52*	31.80 ± 1.77
FS (%)	33.40 ± 1.59	36.45 ± 0.88	45.24 ± 2.05*	53.67 ± 1.01
LV/BW ratio	3.52 ± 0.16	3.20 ± 0.12	3.30 ± 0.31	3.05 ± 0.12
MV PG max (mmHg)	2.69 ± 0.35	1.81 ± 0.35	3.84 ± 0.42	3.23 ± 0.43
MV E (cm/s)	80.21 ± 4.66*	66.50 ± 2.72	96.08 ± 5.28	87.49 ± 5.46
MV A (cm/s)	52.72 ± 3.80*	41.07 ± 2.31	67.03 ± 5.87	65.18 ± 5.87
MV E/A	1.55 ± 0.05	1.66 ± 0.05	1.49 ± 0.07	1.40 ± 0.06

Data are mean values ± SEM. HR, heart rate; LA, left atrial diastolic diameter; LVEDd, left ventricular enddiastolic diameter; EDV, left ventricular enddiastolic volume; FS, fractional shortening; LV/BW ratio, calculated LV weight in relationship to mouse body weight; MV PG max, mitral valve maximal pressure gradient; MV E and MV A, first and second peak of mitral valve flow velocity. *p < 0.05 A_2A_-TG vs. WT (same treatment group).

**Figure 5:**
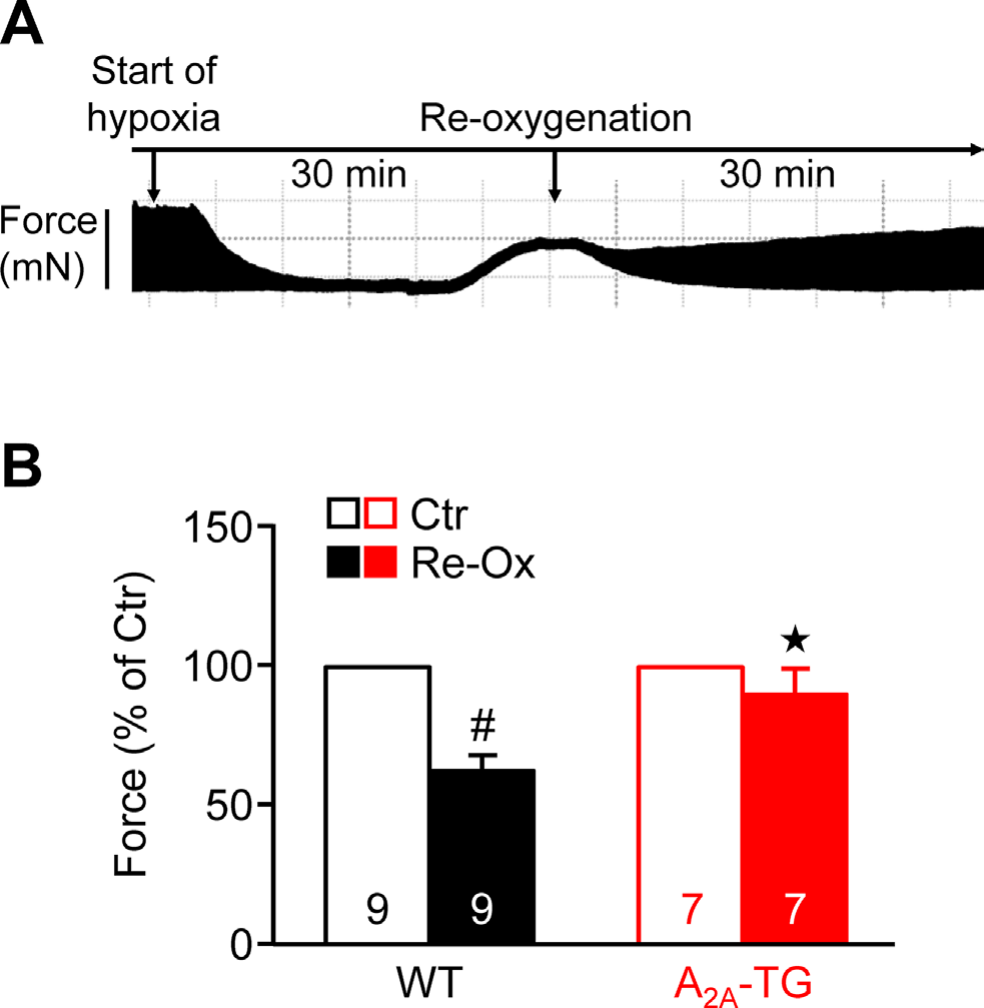
A_2A_-ARs protect atria against hypoxic injury. **(A)** Representative original tracing of a WT left atrium during first hypoxia and re-oxygenation are shown in order to visualize the experimental protocol. **(B)** Force of contraction at the end of the hypoxia re-oxygenation cycle is presented. ^#^*p* < 0.05 vs. Ctr, ^★^*p* < 0.05 vs. WT

Moreover, one could argue that adenosine is known to be released from heart during hypoxia. In order to study the effect of this pathophysiological situation and extending our previous study on ischemia in isolated whole hearts (Boknik et al., 2018), we induced hypoxia in atrial preparations in the organ bath by changing the perfusion gas mixture from 95% O_2_ and 5% CO_2_ to 95% N_2_ and 5% CO_2_. Under these conditions, a rapid decline in force was visible (see **Figure 5A**), which was partially reversible after reoxygenation. Interestingly, atria from A_2A_-TG sustained contractility better than WT (**Figure 5B**).

**Figure 6:**
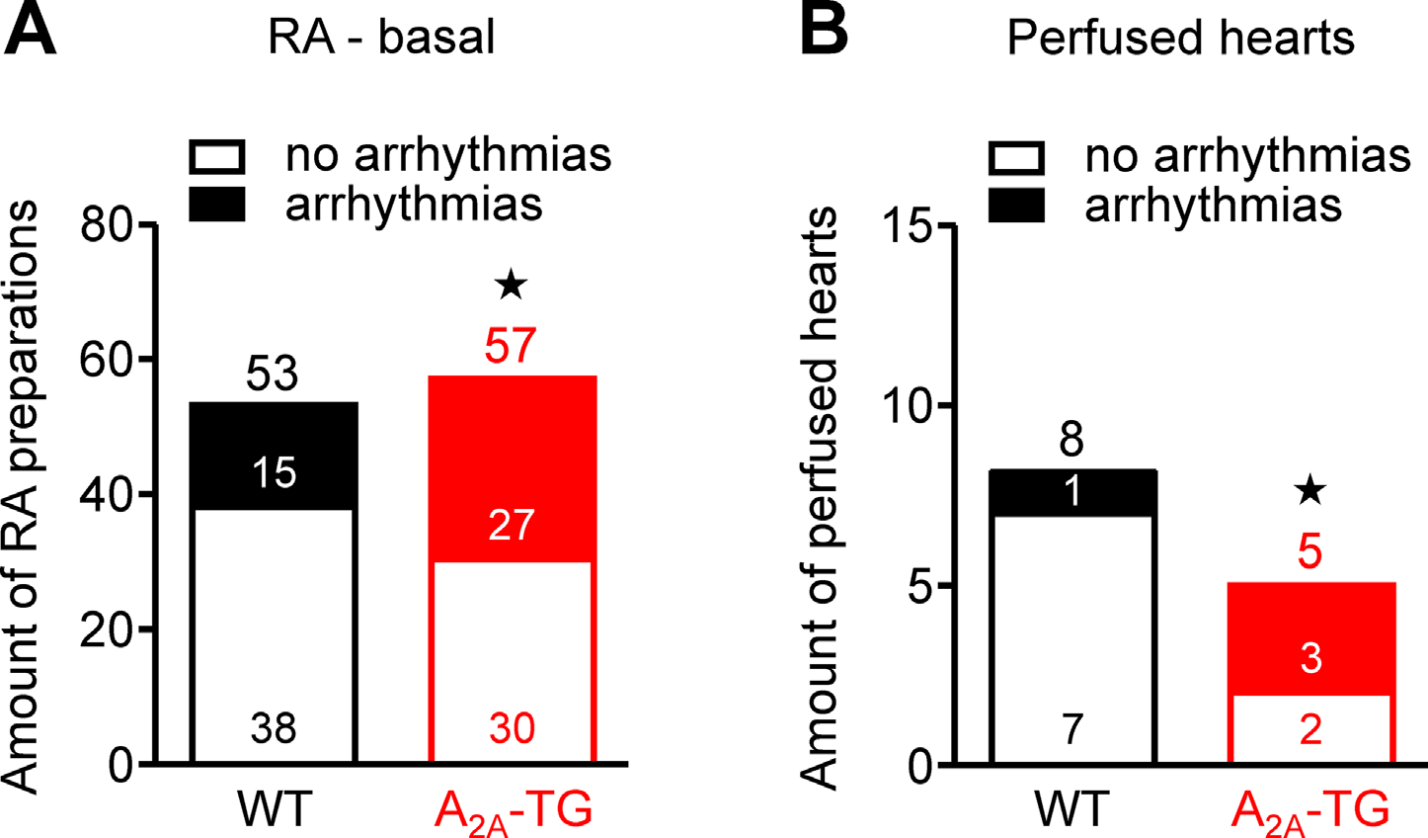
A_2A_-AR increased the incidence of arrhythmias *in vitro*. In isolated spontaneously beating right atrial preparations (RA), we noted a higher propensity to arrhythmias in A_2A_-TG than in WT under basal conditions **(A)**. Also, in isolated perfused hearts, we noted more atrial arrhythmias in A_2A_-TG than in WT **(B)**. ^★^*p* < 0.05 vs. WT (χ^2^-test).

Interestingly, we noted an elevated incidence of CGS 21680-induced arrhythmias in isolated electrically driven left atria (arrhythmias in 2 from 10 in WT and in 6 from 10 in A_2A_-TG, p < 0.05) and in isolated right atrial preparations (arrhythmias in 2 from 16 in WT and in 5 from 17 in A_2A_-TG, p < 0.05). Moreover, even under basal conditions, an increased incidence of arrhythmias was noted in isolated spontaneously beating right atrial preparations from A_2A_-TG compared to WT, and also after β-adrenergic stimulation by addition of isoproterenol to these preparations from A_2A_-TG, the incidence of arrhythmias was higher than in WT (**Figure 6**).

**Figure 7:**
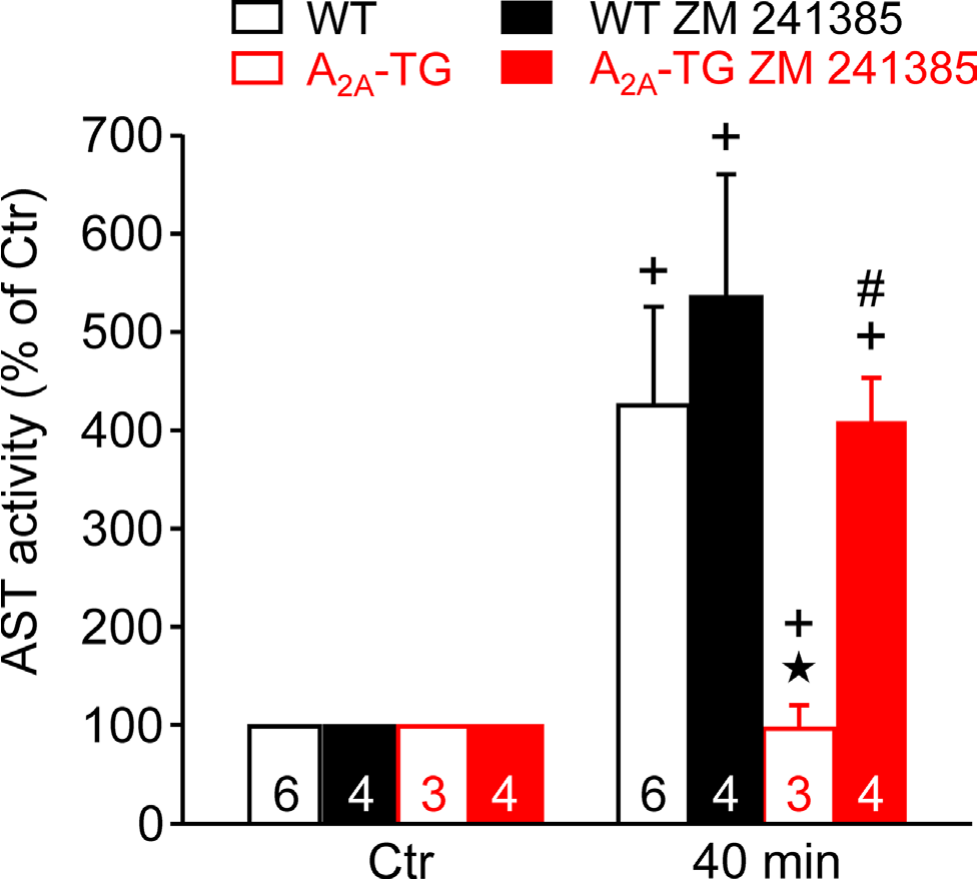
A_2A_-AR protects against cardiac release of AST (aspartate transaminase). Effects of A_2A_-AR after ischemia and reperfusion on the activity of AST are presented. Isolated perfused hearts were prepared from WT and A_2A_-TG and subjected to global ischemia for 20 min, followed by reperfusion. Venous effluates (1 ml) were collected before ischemia (Ctr) and after 40 min of reperfusion. In these effluates, AST enzyme activity was assessed (as % of Ctr). The protective effects of the A_2A_-AR were abolished by the A_2A_-AR antagonist ZM 241385 (1 µM). ^★^*p* < 0.05 vs. WT, + *p* < 0.05 vs. Ctr, ^#^*p* < 0.05 vs. without ZM 241385.

**Figure 8:**
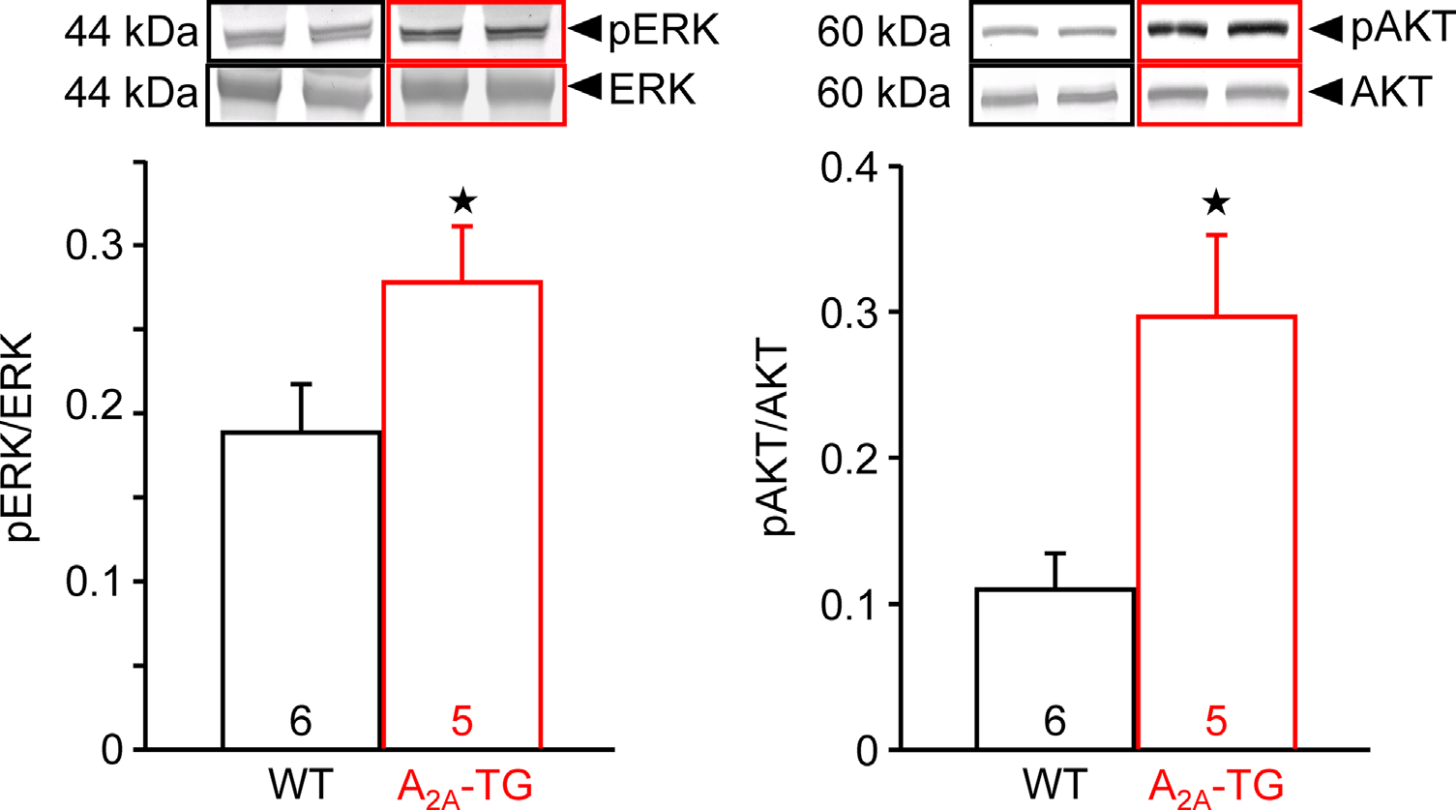
A_2A_-AR exhibited increased the phosphorylation state of proteins in the reperfusion injury salvage kinase (= RISK) pathway. After 20 min ischemia and 40 min reperfusion, isolated perfused hearts (WT and A_2A_-TG) were freeze-clamped. Tissue was homogenized and subjected to electrophoresis and Western blotting. Insets indicate the expression of total ERK and total AKT as well as phosphorylated AKT (pAKT) and phosphorylated ERK (pERK), indicated by arrows and molecular weight in kDa. Ordinates represent the ratio of pAKT to AKT or pERK to ERK. Numbers in brackets indicate the number of hearts studied. ^★^*p* < 0.05 vs. WT.

In our previous study (Boknik et al., 2018), in isolated perfused hearts, we noted depressed contractile response to ischemia. However, the reduction on mechanical function was smaller in hearts from A_2A_-TG than in hearts from WT. Hence, in the present study, we wanted to assess a possible reason for this protective role of A_2A_-ARs biochemically, and therefore, we measured the enzymatic activity of aspartate transaminase (AST; a marker of myocardial damage) in the cardiac perfusate. Indeed, the activity of AST was higher in samples from WT than in samples from A_2A_-TG, and this difference vanished after application of the A_2A_-AR antagonist ZM 241385 (**Figure 7**). Moreover, the phosphorylation states of ERK and AKT were higher in samples from A_2A_-TG than in samples from WT (**Figure 8**).

**Figure 9:**
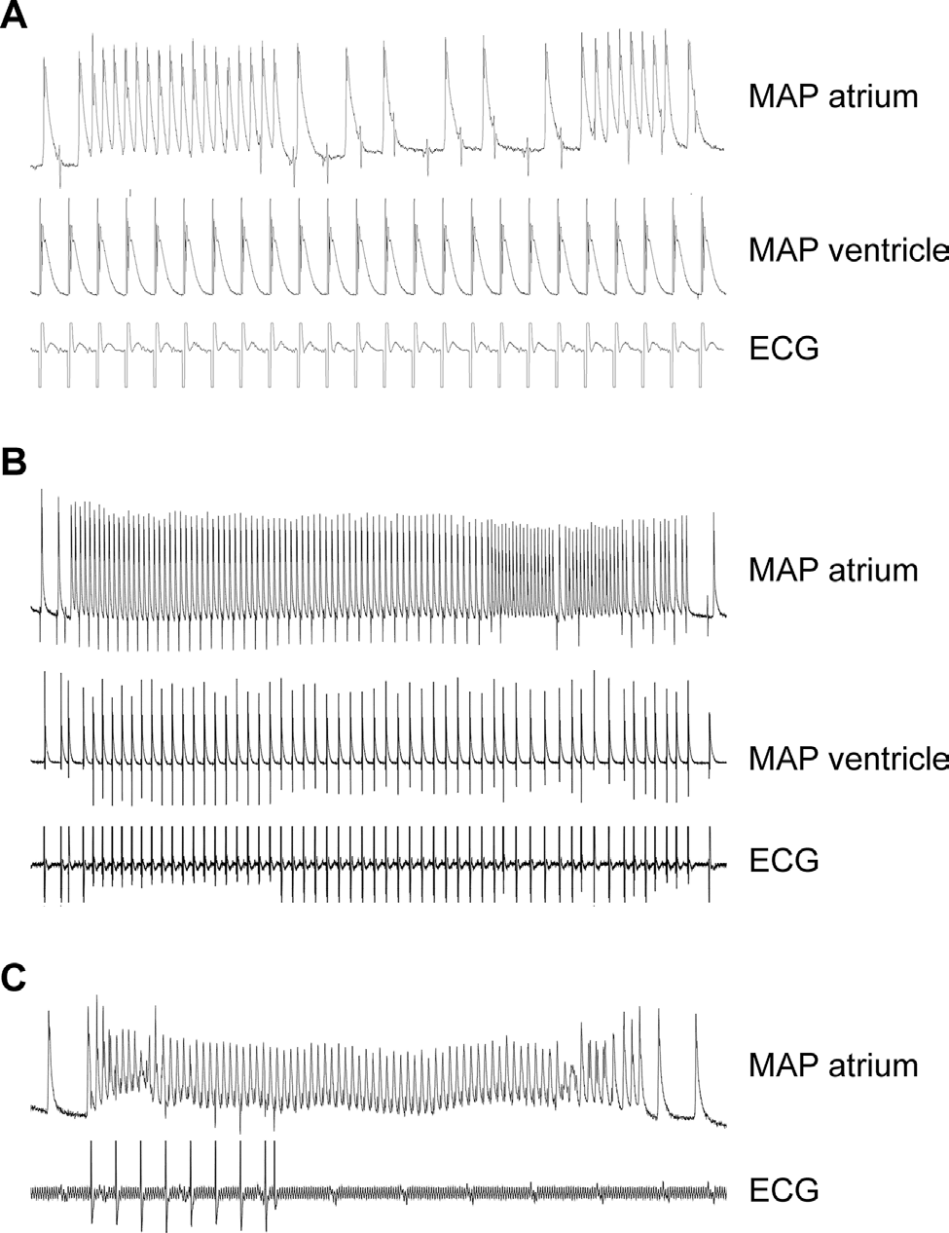
Typical electrical tracings showing different manifestations (atrium and ventricle) of arrhythmias in isolated Langendorff-perfused hearts of A_2A_-TG. Monophasic action potentials (MAP) in atrium (MAP atrium) or ventricle (MAP ventricle) of isolated perfused hearts were established. Non-sustained atrial fibrillation or atrial tachycardia during fixed rate ventricular electrical stimulation were noted in this mouse **(A)**. In **(B)**, a long sustained episode of atrial fibrillation in the same heart could be additionally recorded. In another mouse, a ventricular asystole (see ECG lane) after spontaneous atrial fibrillation **(C)** could be seen. In three out of five A_2A_-TG hearts, atrial and/or ventricular arrhythmias were noted but not in WT (n = 8, χ^2^-test, *p* < 0.05).

In order to study a possible proarrhythmic effect of the A_2A_-AR, we performed monophasic action potential measurements (in isolated perfused hearts). Here, the duration of the monophasic action potential was shortened by CGS 21680 in WT (**Table 2**). Due to the high incidence of arrhythmias, effects of CGS 21680 in A_2A_-TG could not be evaluated under these experimental conditions: in three out of five A_2A_-TG hearts, arrhythmias were noted but not in WT (n = 8, p < 0.05). These arrhythmias manifested as non-sustained atrial fibrillation or atrial tachycardia (**Figures 9A, B**), sometimes leading to ventricular asystole (**Figure 9C**).

**Table 2:** Duration of action potentials in isolated perfused mouse hearts from WT.

		WT CGS21680	Stem CGS21680	WT BASE	Stem BASE	p-values	N WT CGS21680	N WT BASELINE
80	ARD90	23.4	2.2	31.4	1.6	0.073	3	7
	ARD70	11.9	1.0	17.6	1.6	0.027	3	7
	ARD50	8.2	0.9	12.0	1.2	0,065	3	7
	AT	13.1	0.6	13.9	1.4	0.67	3	7
100	ARD90	23.7	0.8	32.5	2.0	0.006	4	7
	ARD70	12.4	0.8	18.9	1.6	0.013	3	7
	ARD50	8.4	0.6	12.8	1.3	0.025	3	7
	AT	13.2	0.4	13.7	1.1	0.699	4	7
120	ARD90	26.3	4.5	33.8	2.4	0.306	3	6
	ARD70	15.6	3.2	20.4	2.0	0.356	3	6
	ARD50	10.6	2.3	13.7	1.6	0.41	3	6
	AT	13.9	1.2	13.8	1.3	0.957	3	6

Cycle length of 80, 100, and 120 ms (first column) under basal conditions (basal) and after application of 1 µM CGS 21680.

**Figure 10:**
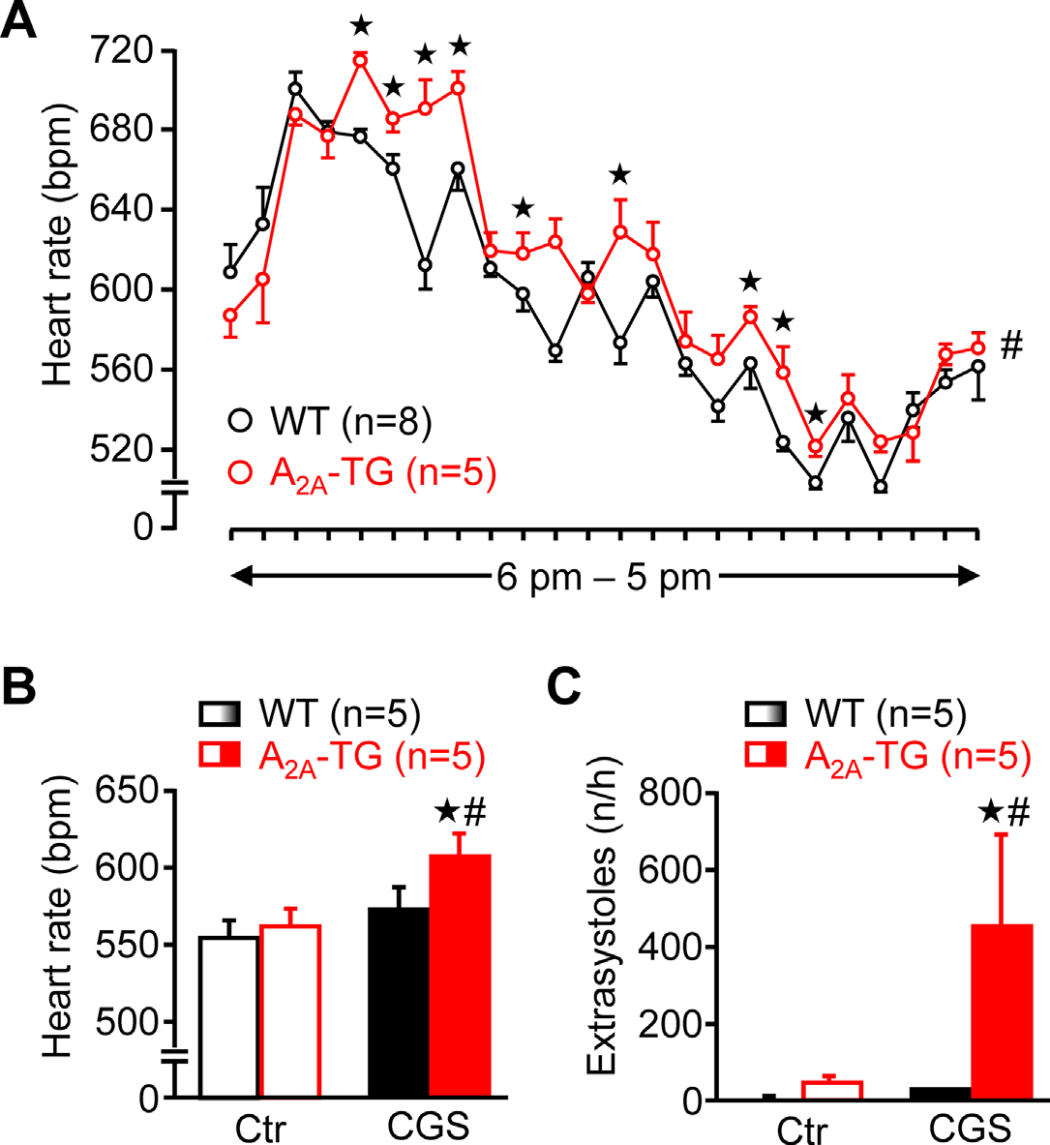
A_2A_-AR expression per se can increase heart rate *in vivo*. Heart rate (HR) of freely moving WT (n = 8) and A_2A_-TG mice (n = 5) was telemetrically recorded within 24 h **(A)**. Mean HR is plotted in hourly intervals. ^★^*p* < 0.05 vs. WT. ^#^*p* < 0.05 analysis of variances of the whole curves between A_2A_-TG and WT. Under resting conditions, ECGs (Ctr) were telemetrically recorded, and 1 h after CGS 21680 injection (40 µg kg^−1^), all arrhythmic events were assessed in the following hour. CGS 21680 increased under these conditions the heart rate in A_2A_-TG but not WT mice **(B)**. Moreover, CGS 21680 injection was accompanied by a higher incidence of ventricular extrasystoles in A_2A_-TG than in WT **(C)**. ^#^*p* < 0.05 vs. Ctr, ^★^*p* < 0.05 vs. WT.

Using a more physiological set up, we studied the role of A_2A_-ARs in the heart using telemetric ECG recordings of freely moving mice. Under these conditions, the spontaneous mean heart rate was at several time points higher in A_2A_-TG than in WT during a 24 h period (**Figure 10A**). Besides these baseline long-term ECGs, telemetric ECGs after i.p. drug injection were also obtained. In A_2A_-TG, CGS 21680 induced a fast and pronounced heart rate increase (**Figure 10B**), and CGS 21680 induced more ventricular extrasystoles in A_2A_-TG than in WT under these conditions (**Figure 10C**).

## Discussion

We noted several bands on Western blots of hearts from A_2A_-TG when probing the blots with an antibody raised against a peptide from the sequence of the A_2A_-AR. Lower molecular bands that occur only in samples from A_2A_-TG but not in WT and which vanished when preincubated with a blocking peptide are of smaller molecular weight and may represent proteolytic fragments of the receptor. When carefully looking at the blot, at least one specific higher molecular weight band is seen. We speculate here that this might be a dimeric form of the receptor. Dimeric forms of A_2A_-AR have been detected in cell cultures of transfected cells and have been claimed to represent the functional form of the receptor on the cell surface (HEK-293 cells: Burgueño et al., 2003).

It is relevant that we noted a positive inotropic effect of CGS 21680 in human right atrial samples (which to the best of our knowledge has not been reported before), as this underscores the functionality of this receptor in the human heart and more specifically a similarity (and comparability) of our atrial preparations from transgenic mice with overexpression of the A_2A_-AR and at least the human atrium.

**Figure 11:**
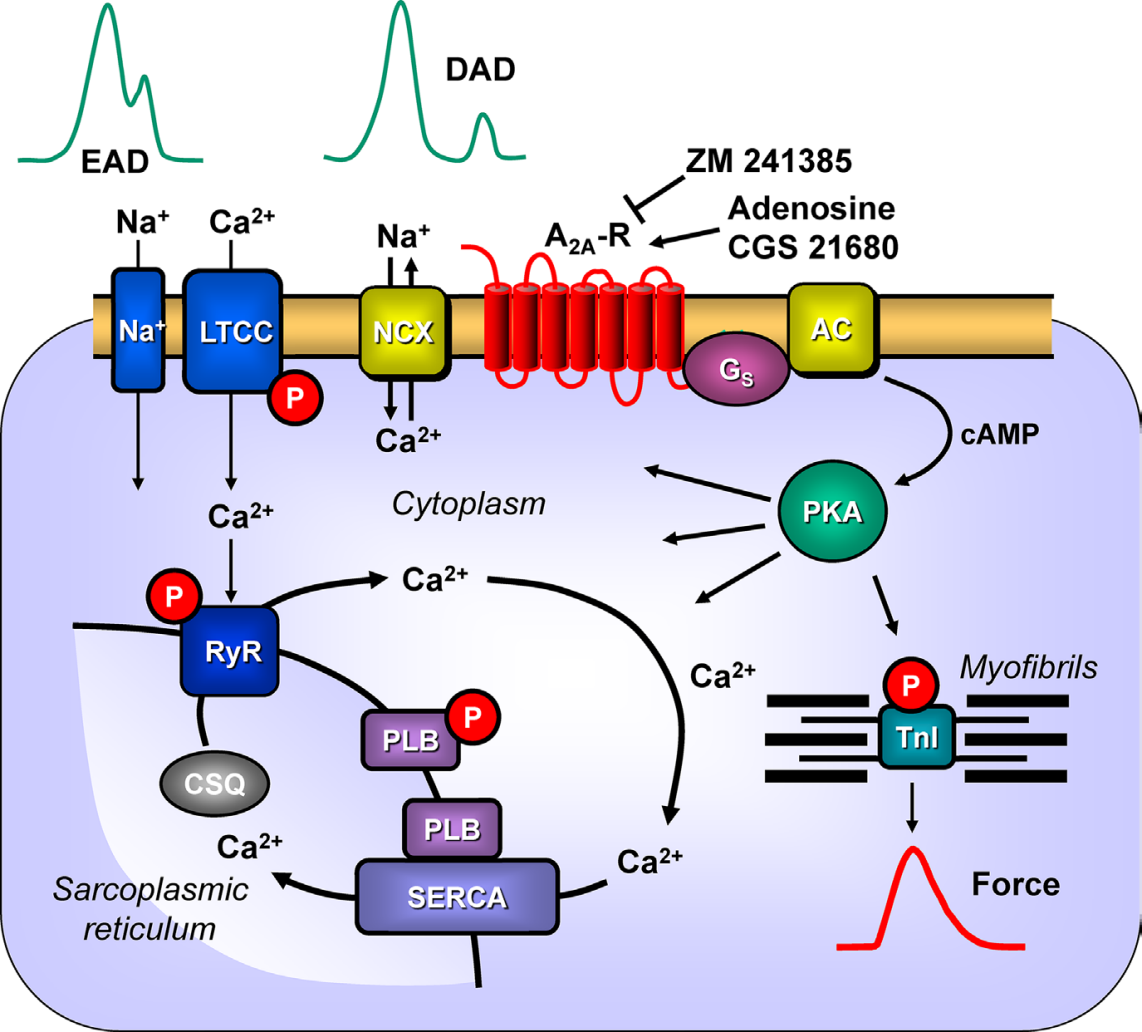
Possible signaling pathways through A_2A_-ARs in cardiac myocytes facilitating arrhythmias. Heptahelical A_2A_-ARs are activated physiologically by adenosine. Moreover, the more selective agonist CGS 21680 activates the A_2A_-ARs, whereas ZM 241385 will block the receptor. Activation of A_2A_-ARs leads *via* stimulatory G-proteins (Gs) to the increased activation of adenylyl cyclase (AC) and generation of cAMP. This activates the cAMP-dependent protein kinase (PKA). PKA is known to phosphorylate the inhibitory subunit of troponin (TnI) to hasten relaxation and phospholamban (PLB), which de-inhibits the activity of the sarcoplasmic reticulum ATPase (SERCA). We speculate that under these conditions A_2A_-AR activation will also lead to the phosphorylation of the cardiac RYR2 and the L-type calcium channel (LTCC). Opening of the RYR2 releases Ca^2+^ that can bind to troponin C and initiate muscle contractions. Typically, increased opening of RYR2 would lead to high concentrations of Ca^2+^in the vicinity of the sarcolemmal sodium calcium exchanger (NCX). This will lead to loss of Ca^2+^from the cell and sodium influx. As the NCX is electrogenic, this will lead to depolarization of the cell to delayed afterdepolarizations (DAD) and subsequent arrhythmias. Moreover, enhanced function of sodium channels (Na^+^) or through LTCC would facilitate early afterdepolarizations (EAD). Both EAD and DAD are known to lead to mechanical arrhythmias in the mammalian heart. In addition, there is growing evidence for the involvement of AKT and ERK not only in protection from hypoxia but also on an arrhythmogenic effect.

Previously, we had shown (Boknik et al., 2018) that CGS 21680 increased the cAMP content and cAMP-dependent phosphorylation of the proteins phospholamban and the inhibitory subunit of troponin in A_2A_-TG but not in WT. Here, we extended the functional relevance of phospholamban phosphorylation (**Figure 11**) by showing that CGS 21680 also increased cytosolic Ca^2+^ and contractility in isolated electrically driven ventricular cardiomyocytes from A_2A_-TG but not in WT. The increase in Ca^2+^ transients is also a plausible explanation why A_2A_-TG can show arrhythmias in atrium and ventricle, conceivably because A_2A_-ARs are stimulated by endogenously produced adenosine (**Figure 11**).

The fact of an increased incidence of CGS 21680-induced arrhythmias in isolated atrial preparations of A_2A_-TG is consistent with observations in humans. In detail, patients with clinically known atrial fibrillation and increased susceptibility for altered Ca^2+^ handling showed up-regulated A_2A_-AR mRNA expression. In isolated atrial cardiomyocytes from these patients, an increased incidence of spontaneous SR calcium releases was noted after addition of adenosine or CGS 21680 (Llach et al., 2011a).

Contractile function in hypoxia was studied to assess possible involvement of endogenous adenosine formed e.g. from ATP during lack of oxygen in the atrial cardiomyocytes. Interestingly, force reappeared better in A_2A_-TG than WT (as reported before Boknik et al., 2018). Overexpression of the A_2A_-AR apparently protects against cardiac damage because the enzymatic activity of AST, a marker of the inability of the sarcolemma to contain ingredients within the cell, was only increased in WT but not in A_2A_-TG. This protection was in all probability mediated by A_2A_-ARs as this protective effect in cardiac preparations from A_2A_-TG was abolished by applying the A_2A_-AR antagonist ZM 241385. The protective effect might involve the mitochondria as the phosphorylation state of AKT (a phosphoprotein linked to mitochondrial function) was increased to a higher extent during reperfusion in hearts from A_2A_-TG than in WT. A role of active AKT and/or ERK1/2 in cardiac protection but also generation of arrhythmias has been reported by others before (Cheng et al., 2016;Ezeani and Elom, 2017; Hu et al., 2019) and underscores a putative role of these pathways in our model system.

Extending the data on isolated tissue, we confirmed in the living animal the positive chronotropic effect of A_2A_-AR overexpression alone and its stimulation by an A_2A_-AR selective agonist in the living animal in telemetric ECG measurements. We think it is of special relevance that after A_2A_-AR stimulation, we could detect an enhanced incidence of arrhythmias in living animals, indicating that the proarrhythmic effect of A_2A_-AR expression might be so strong that vagal or other neural compensatory mechanisms cannot overcome it and we predict the same might apply for humans.

An explanation of the higher beating rates noted in right atrial preparations of 30-week-old A_2A_-TG (Boknik et al., 2018) and also in freely moving mice might be an increased basal level of cAMP production due to the overexpressed A_2A_-AR.

Mouse models with cardiac-restricted overexpression of the human A_2A_-AR have also been reported before by others. In an earlier work, the group of Feldman studied in a mouse model with constitutive overexpression of the A_2A_-AR the functional interaction of coexpression of A_1_- with A_2A_-ARs *in vivo* (Chan et al., 2008). Later, they studied the protective role of A_2A_-AR by inducible overexpression of the A_2A_-AR in the mouse heart in a pressure overload model (aortic banding: Hamad et al., 2012) or the effect of the A_2A_-AR on adriamycin-induced cardiotoxicity (Hamad et al., 2010). However, we used our model to begin a first study on a putative proarrhythmic role of A_2A_-AR overexpression in the mouse heart (Boknik et al., 2018).

The question then arises whether this model has any (patho)-physiological relevance. For example, A_2A_-AR agonists like regadenoson are clinically used to detect latent ischemia in patients (Cerqueira et al., 2008). Hence, it is conceivable that A_2A_-AR activation could lead to arrhythmias at least in those patients who already have higher levels of A_2A_-ARs. Recently, A_2A_-AR stimulation in isolated human atrial myocytes has been shown to promote irregularities in calcium transients like spontaneous calcium ion waves (Molina et al., 2016). Spontaneous Ca^2+^ release has been reported to initiate atrial fibrillation in human atrial myocytes (Hove-Madsen et al., 2004; Llach et al., 2011b; Voigt et al., 2012).

From a mechanistic point of view, our study adds to our knowledge. In patients, it is usually only possible to describe which gene alterations are found in e.g. atrial fibrillation. For instance, there is a wealth of clinical information that the expression of A_2A_-ARs is increased in arrhythmias, but likewise, the expression of angiotensin II-receptor, IP_3_-receptors, opioid receptors, and 5-HT_4_-receptors was reported by others to be elevated (Goette et al., 2000; Yamda et al., 2001; Lendeckel et al., 2005; Boldt et al., 2006; Gassanov et al., 2006; Lezoualc’h et al., 2007). Hence, it is not clear which of the overexpressed receptors in human atrium is more relevant for the genesis of arrhythmias. However, the present work clearly indicates that A_2A_-AR overexpression alone is able to increase the propensity to arrhythmias.

Many years ago, we reported an increased A_2A_-AR mRNA expression in failing human ventricles compared to nonfailing ventricles (Stein et al., 1998). Therefore, we speculate that A_2A_-AR may contribute to arrhythmias in end stage human heart failure. Another open question is whether the increase in A_2A_-ARs is the cause or the results of atrial fibrillation in humans. A_2A_-AR activation is used in the clinic to assess the vasodilatory capability of coronary arteries for nuclear magnetic studies (Palani et al., 2011). Moreover, adenosine is used to treat supraventricular arrhythmias. Some of the untoward effects of adenosine in the context (flushing, hypotension) have been attributed to the action of vasodilatory A_2A_-ARs (review: Szentmiklosi et al., 2015). Finally, istradefylline and tozadenant, A_2A_-AR antagonists, are new compounds used in patients to treat Morbus Parkinson (Hauser et al., 2014; Schapira et al., 2014; Adams et al., 2015). Their potential cardiac side effects have been partially studied in healthy volunteers (Wang et al., 2013). Other A_2A_-AR antagonists have been studied clinically for treatment of melanoma (Adams et al., 2015). One could speculate from our data that these compounds might exert antiarrhythmic effects in some patients.

## Conclusions

Based on our recent work (Boknik et al., 2018), we further characterized a transgenic mouse with cardiomyocyte-specific overexpression of the A_2A_-AR. It is noteworthy that the A_2A_-AR shows a Janus-faced behavior: protection against ischemic damage but induction of cardiac arrhythmias. Our main new finding is that this overexpression alone and receptor activation by A_2A_-AR agonists increase the occurrence of cardiac arrhythmias in the mammalian heart. It is tempting to speculate that A_2A_-AR antagonists might be useful antiarrhythmic agents in selected patients in the future.

## Data Availability

All datasets generated for this study are included in the manuscript/supplementary files.

## Ethics Statement

The animal study was reviewed and approved by Animal welfare committee of the University of Münster, Germany.

## Author Contributions

KD, JE, SG-W, LFa, LFo, BH, HT, and UK performed the research. PB, FM, WS, and JN designed the research study. PB, PK, LFa, NZ, UK, and JN analyzed the data. PB, UG, and JN wrote the original draft. PB and UG performed the visualization. PB, FM, WS, NZ, UG, and JN reviewed and edited the paper.

## Funding

We acknowledge the financial support within the funding program Open Access Publishing by the German Research Foundation (DFG).

## Conflict of Interest Statement

The authors declare that the research was conducted in the absence of any commercial or financial relationships that could be construed as a potential conflict of interest.
